# Expression of the human microRNA miR 29a in an Indian cohort of HIV patients

**DOI:** 10.1186/1471-2334-14-S3-O9

**Published:** 2014-05-27

**Authors:** Rakesh Dey, Kartik Soni, Shanmugam Saravanan, Pachamuthu Balakrishnan, Vikram Kumar, Vinod Scaria, Beena Pillai, Suniti Solomon, Samir K Brahmachari

**Affiliations:** 1 CSIR institute of Genomics and Integrative Biology, Mathura Road, Delhi 110020, India; 2 YR Gaitonde Centre for AIDS Research and Education, VHS Campus, Rajiv Gandhi Road, Taramani, Chennai 600113, India

## Background

The events during host virus interaction pave the way to differential outcomes in HIV-1 disease progression rates. We have earlier shown that HIV-*1Nef* gene harbors target sites for the human microRNA, miRNA 29a/b. Over expression of miR 29a/b could reduce virus levels by targeting the *Nef* gene. We hypothesized that differential expression of the microRNA maybe a protective factor in AIDS progression.

## Methods

In a cohort of 75 HIV-1 infected individuals, who show differential disease progression, classified as (1) Long Term Non Progressors (LTNPs), (2) Regular Progressors and (3) Rapid Progressors. We isolated RNA and DNA from PBMCs collected from these individuals and healthy controls. We quantified miR 29a expression through Real Time PCR and performed statistical correlation of the miRNA levels with disease progression rate. We further analyzed miR 29a promoter and coding region from selected patients.

## Results

Patients, including various sub classes like LTNPs, Regular Progressors and Rapid progressors, had higher miR 29a levels than healthy controls. A small subset of patients had unusually high levels of miR 29a. Three of these individuals were LTNPs while others were classified as regular progressors.

## Conclusion

The absence of known genetic causes of non progression like the CCR5 delta mutation, in this patient cohort suggests that there are novel mutations that confer protection. The absence of healthy control individuals with high miR 29a expression suggests that the induction of miRNA maybe happen in response to infection. However, larger sample sizes are required to confirm this. We conclude that miR 29a expression maybe one of the factors that in certain backgrounds provides a better prognosis.

